# Recurrence rate of intervertebral disc disease in surgically treated French Bulldogs: a retrospective study (2009–2019)

**DOI:** 10.1186/s13028-023-00667-0

**Published:** 2023-02-02

**Authors:** Dominique Leu, Beatriz Vidondo, Veronika Stein, Franck Forterre

**Affiliations:** 1 Department of Clinical Veterinary Medicine, Division of Surgery, Vetsuisse Faculty, University of Bern, Länggassstrasse 128, 3001 Bern, Switzerland; 2 Departement of Clinical Research and Public Health (SCR-VPH), Vetsuisse Faculty, University of Bern, Schwarzenburgstrasse 161, 3097 Köniz, Switzerland; 3 Department of Clinical Veterinary Medicine, Division of Neurology, Vetsuisse Faculty, University of Bern, Länggassstrasse 128, 3001 Bern, Switzerland

**Keywords:** Epidemiology, French Bulldog, Intervertebral disc herniation, Recurrence

## Abstract

**Background:**

Intervertebral disc disease (IVDD) is a common diagnosis and well-investigated pathological condition in French Bulldogs with neurological deficiencies. However there is currently only one recently published retrospective descriptive study looking for recurrence rates of IVDD in French Bulldogs. Medical reports of French Bulldogs with a first episode of IVDD and surgical treatment were evaluated and reviewed for clinical signs of recurrence. Risk factors for Total-Recurrence, Cervical- and Thoracolumbar-Recurrence were evaluated by means of logistic regression models. The aim of this study was to assess frequency and risk factors associated with the recurrence of IVDD in French Bulldogs.

**Results:**

One hundred twenty-seven French Bulldogs with a first episode of IVDD and surgical treatment were evaluated. 52.7% (67/127) of these patients showed signs of recurrence. The recurrence rate in the cervical spine was slightly lower (47%) compared to the thoracolumbar spine (56.6%). A significant association with recurrence could be found for the factor age: French Bulldogs with a first episode of IVDD ≤ 3 years seem to be prone for Total-Recurrence (P = 0.002) and Cervical-Recurrence (with ORs ranging from 0.02 to 0.03 for patients older than 3 years). 50% of the recurrences (median) occurred within the first 12 month after the first episode of IVDD.

**Conclusions:**

Recurrence of IVDD can be expected in more than half of French Bulldogs affected by IVDD. Especially young French Bulldogs are prone for recurrence in cervical spine. Almost every fourth patient with IVDD suffers from a recurrence within 12 months. Future dog owners should be informed about the risk of IVDD and the early onset of recurrences in French Bulldogs.

## Background

The French Bulldog is a common dog breed gaining in popularity each year [1]. Intervertebral disc disease (IVDD) is the most common neurological disorder (in 45.5% of cases) in French Bulldogs [2]. As they belong to the chondrodystrophic dog breeds, they are prone for degenerative changes of the intervertebral disc (IVD) [3]. Compared to other dog breeds, French Bulldogs are presented at younger ages with a first episode of IVDD and are affected more commonly by IVDD in the cervical spine [2]. The most common cause for recurrence of IVDD is the development of a second episode of IVDD at other intervertebral disc spaces (IVDS) [4–6]. Different studies on different dog breeds with surgical treatment after IVDD showed recurrence rates of 0–17% in the cervical spine [7–9] and 2.7–41.7% in the thoracolumbar spine [4, 7, 10–13]. A recent study by Kerr et al. [14] focusing on French Bulldogs only showed a recurrence rate of 51% after decompressive surgery of cervical and thoracolumbar IVDD.

The aim of this study is to characterize the recurrence of IVDD in French Bulldogs more precisely with focus on the frequency as well as the association of different risk factors with Total-Recurrence, Cervical- and Thoracolumbar-Recurrence separately.

## Methods

The data were collected using the clinical reports (Polypoint AG, Gümligen, Switzerland, version 18.2.060, 2018) of the entry exam, neurological examination and radiological reports of all French Bulldogs presented for signs related to IVDD to the Small Animal Clinic of the Vetsuisse Faculty, University of Bern, Switzerland between 2009 and 2019. The first episode of IVDD in all French Bulldogs included in this study had to be confirmed by magnetic resonance imaging (MRI) examination. Patients were excluded if they were previously diagnosed with spinal diseases other than IVDD. Only surgically treated patients were included in this study. The minimal follow up time comprised 6 months. If the dogs were not presented for re-evaluation in the hospital, the owners were contacted by phone and asked about recurring clinical signs in their dogs. Recurrence was defined as additional episodes of IVDD that occurred after recovery from a first episode. A recurrence was considered as confirmed if the diagnosis was reached by MRI examination, whereas it was considered suspected if it was diagnosed based on clinical signs highly indicative of IVDD (acute onset of spinal hyperesthesia and/or neurological deficits compatible with a single spinal lesion) without further diagnostics [14]. Patients in the sample had to become either free of neurological signs or regain an ambulatory status with mild residual deficits [14] Recovery was therefore defined as improvement of neurological status after therapy of the first episode according to the classification of the neurological grades [15, 16]. Patients without improvement of non-ambulatory status and with lack of information concerning recurrence of clinical signs were excluded. In contrast, patients with missing data for a single specific risk factor for recurrence were kept in the sample. For patients with more than two episodes of IVDD, only the first and second episodes attributed to IVDD were evaluated.

### Outcomes and risk factors

We investigated the Total-Recurrence of IVDD (yes/no) and the recurrence calculated for cervical- and thoracolumbar IVDD separately (yes/no) after surgery of a first episode in the particular spinal segment. The following risk factors were evaluated for their potential influence on the recurrence rate: Age, gender, body weight, severity (neurological grade), anatomical localization of the first IVDD, the existence of vertebral malformations (yes/no), the existence of intervertebral disc degeneration (IVDd, yes/no) and the use of prophylactic fenestration during surgery of the first episode of IVDD (yes/no).

Age was recorded at presentation of their first episode of IVDD in years. All patients were then categorized into four classes according to quartiles (25% = 3 years, 50% = 4 years, 75% = 5 years). Thus, patients were grouped as follows: ≤ 3 years (0–47 month), 4 years (48–59 month), 5 years (60–71 month)  ≥ 6 years (72–120 month).

Body weight was evaluated at presentation of first episode of IVDD in kilogram and evaluated as continuous variable.

The severity of neurological signs was clinically determined at first presentation with IVDD and assigned to one of the five different grades (according to the modified Frankel score) in the thoracolumbar spine [15, 17]: grade 1—hyperesthesia without neurological deficits, grade 2—ambulatory paresis, grade 3—non-ambulatory paresis and ataxia, grade 4—paralysis, and grade 5–paralysis without deep pain sensation. For the cervicale spine a grading system according to Ryan et. al. [16, 18] was used: 1—neck pain only, 2—proprioceptive deficits or forelimb lameness, 3—ataxia, 4 – ambulatory paresis, 5—non-ambulatory paresis with intact nociception. As two different grading systems were used neurological severity grade was not evaluated for Total-Recurrence.

Localization of the affected intervertebral disc space (IVDS) at the first and second episode of IVDD was analyzed and categorized into cervical (C1-T2) or thoracolumbar (T3-S3) IVDD.

The locations of vertebral malformations (wedge vertebra, butterfly vertebra and thoracolumbar transition vertebra,) were recorded using MRI imaging and categorized as being less than or more than 2 IVDS away from the original IVDD lesion. Patients with no vertebral malformation were categorized into a third group.

Based on MRI findings during the first episode of IVDD, IVD of the investigated spinal segment were categorized as degenerated or not-degenerated based on the signal intensity on midsagittal T2 MRI images [19]. “Non-degenerated IVD” were defined as having a homogenous T2 hyperintense signal in the nucleus pulposus, whereas a “degenerated IVD” had a loss of T2 hyperintense signal. The MRI studies were blinded and assessed by board certificated radiologists.

All patients were treated with decompressive surgery with removal of the extruded disc material for their first episode of IVDD by board-certificated surgeon, neurologist or residents of each department. According to the surgical reports all patients were examined for additional prophylactic disc fenestrations to prevent further disc extrusions. Patients were categorized into two groups based on receiving additionally a disc fenestration or not.

### Statistical analysis

The data were analyzed using the statistics software R (version 3.6.1 [20]) and Rcmdr package. To assess the influence of the risk factors on recurrence of IVDD, logistic regression models investigating the effect of different risk factors on recurrence of IVDD were estimated for all patients together, as well as for patients with cervical and thoracolumbar IVDD separately. The data were analyzed using multivariable logistic regression analysis.

## Results

One hundred and eighteen out of 245 patients had to be excluded due to incomplete reports or lack of neurological improvement after first episode of IVDD. Consequently, 127 French Bulldogs with a first episode of IVDD were included of which 67 dogs showed a recurrence (52.7%). Of these dogs, 31/67 (46.2%) were reexamined by MRI (confirmed) whereas 36/67 (53.7%) dogs were diagnosed by clinical signs only (suspected). Mean time to recurrence evaluated in 67 patients was 18.7 months with a median of 12 months (range 1–82 months). The mean observation period was 42 months (3.5 years) and the median was 32.5 months (2.7 years) with a range of 6 month to 9 years. 35/67 (52.2%) recurrences occurred within the first 12 months after the first episode of IVDD. During the second year following the first IVDD, only 14/72 (19.4%) of the patients suffered a recurrence. In the following years, the recurrence rate dropped further to 12/72 (16.6%), 5/72 (6.9%) and 2/72 (2.7%), respectively. A description of the patients in our sample is displayed in Table 1. Risk factors are presented in Table 2.

**Table 1:** Summary statistics of patients with intervertebral disc disease (IVDD)

	IVDD				Recurrence cervical		Recurrence thoracolumbar	
	1st (n)	Recurr (n)	Sus (n)	Con (n)	1st (n)	Recurr (n)	1st (n)	Recurr (n)
N	127	67	36	31	51	24	76	43
Gender	127	67	36	31	51	24	76	43
Male	73	38	18	20	34	16	39	22
Female	54	29	18	11	17	8	37	21
Weight	127	67	36	31	51	24	76	43
Age	124	65	35	30	48^a^	22^a^	76^b^	43^b^
≤ 3 years	46	31	16	14	51^a^	24^a^	33^b^	20^b^
4 years	22	12	9	3	26^a^	15^a^	12^b^	8^b^
5 years	29	14	6	8	8^a^	4^a^	19^b^	10^b^
≥ 6 years	27	8	4	5	6^a^	2^a^	12^b^	5^b^
Localization	127	67	36	31				
Cervical	51	24	12	12				
Thoracolumbar	76	43	24	19				
Severity grade	127	67	36	31	51^a^	24^a^	76^b^	43^b^
1					30^a^	18^a^	5^b^	2^b^
2					11^a^	4^a^	19^b^	12^b^
3					9^a^	1^a^	30^b^	19^b^
4					1^a^	1^a^	19^b^	8^b^
5					0^a^	0^a^	3^b^	2^b^
Malformation	127	67	36	31	51	24	76	43
Less than 2 IVDS	18	11	8	3	1	1	17	10
More than 2 IVDS	28	16	9	7	3	3	25	13
No malformation	81	40	19	21	47	20	34	20
Disc degeneration	127	67	36	31	51	24	76	43
Degenerated disc	99	50	29	22	37	14	62	36
No degenerated disc	28	17	7	9	14	10	14	7
Disc fenestration	117	67	36	31	51	24	76	43
Disc fenestration	17	7	34	26	4	1	11	4
No disc fenestration	110	60	2	5	47	23	65	39

*1st,* first episode of IVDD; *Recurr*, recurrence

*Sus,* suspected recurrence of IVDD; *Con,* confirmed recurrence of IVDD

a = Grading to Ryan et al. [16], b = grading to Modified Francel score [15]

**Table 2:** Results of the multivariable logistic regression models

	Total recurrence (yes/no)		Recurrence cervical (yes/no)		Recurrence thoracolumbar (yes/no)	
	P-value	OR (95 % CI)	P-value	OR (95 % CI)	P-value	OR (95 % CI)
Gender						
Female	0.66	1.2(0.5–3.2)	0.28	0.2(0.02–2.6)	0.86	1.1(0.3–3.8)
Weight (kg)	0.12	1.2(0.9–1.5)	0.32	1.4(0.7–2.7)	0.20	1.2(0.9–1.6)
Age						
4 years	0.26	0.5(0.1–1.7)	0.05*	0.03(0.0009–0.8)	0.75	1.3(0.2–7.8)
5 years	0.06	0.3(0.1–1.2)	0.02*	0.02(0.0005–0.4)	0.43	0.5(0.1–2.4)
≥ 6 years	0.002**	0.2(0.04–0.5)	0.01**	0.03(0.001–0.4)	0.18	0.3(0.06–1.6)
Localisation						
Thoracolumbar	0.3	1.9(0.6–6.4)				
Severity grade						
2			0.28	0.2(0.01–2.6)	0.19	4.4(0.5–49.7)
3			0.28	0.2(0.08–3.1)	0.27	3.1(0.4–29.7)
4			0.08	0.05(0.0006–0.9)	0.89	1.2(0.1–11.7)
5			0.99	–	0.65	2.1(0.08–88.9)
Malformation						
More than 2 IVDS	0.9	0.9(0.2–4.0)	0.99	–	0.92	0.9(0.18–4.6)
No malformation	0.49	0.6(0.2–2.4)	0.99	–	0.78	1.2(0.3–5.8)
Disc degeneration						
No degenerated disc	0.79	1.1(0.4–3.2)	0.15	6.4(0.6–11.4)	0.25	0.4(0.1–1.7)
Disc fenestration						
No fenestration	0.18	0.5(0.1–1.4)	0.34	6.0(0.2–41.1)	0.10	0.3(0.06–1.3)

- Not estimable due to lack of data

^*^Significance level 0.05 (in bold)

**Significance level 0.01 (in bold)

### Gender

Similar recurrence rates were observed in female and male dogs and thus no significant association with recurrence of IVDD could be detected.

### Body weight

Patients had an average body weight of 12.8 kg with a median of 13.0 kg at the first episode of IVDD. There was no significant influence with recurrence of IVDD:

### Age

The mean age at the first episode of IVDD was 4.3 years with a median of 4.0 years, with 46/124 (37.1%) of the IVDD occurring in the first 3 years of life. French Bulldogs ≤ 3 years had the highest recurrence rate with 47.7% (31/65). Patients ≥ 6 years were significantly less likely for recurrence than patients ≤ 3 years (OR = 0.2, P = 0.002). When looking at the cervical spine only, this effect is even more pronounced: Age seems to be a protective factor, with patients > 3 years being at lower risk for recurrence (4 years OR = 0.03 P = 0.05; 5 years OR = 0.02 P = 0.02; ≥ 6 years OR = 0.03 P = 0.01) based on multivariable analysis. By contrast, no significant association was found in the thoracolumbar spine.

### Localization

59.8% (76/127) of first episodes of IVDD were located in the thoracolumbar spine, 56.6% (43/76) of these patients showed signs of recurrence. 51/127 of patients (41.1%) suffered a first episode of IVDD in the cervical spine and 43.6% (24/55) developed a recurrence. There was no significant association of localization of the first episode and the recurrence of IVDD. As shown in Table 3, most frequently affected IVDS at first episodes in the thoracolumbar spine was L3-L4 (35.8%) with a recurrence rate of 62.5%. In the cervical spine, the most frequently affected IVDS was C3-C4 (56.8%) with a recurrence rate of 62.1%.

**Table 3:** Localization of 1st episode of intervertebral disc disease (IVDD)

IVDS	1st (n)	Recurr (n)	Recurr (%)	IVDS	1st (n)	Recurr (n)	Recurr (%)
Total	76	43	56.6	Total	51	24	47
T8-T9	1	0	0	C2-C3	8	3	37.5
T11-T12	2	0	0	C3-C4	29	18	62.1
T12-T13	7	4	57.1	C4-C5	9	2	22.2
T13-L1	12	6	50	C5-C6	5	1	20
L1-L2	8	6	75				
L2-L3	10	7	70				
L3-L4	24	15	62.5				
L4-L5	9	5	55.5				
L4-L6	3	0	0				

*1st*, first episode of IVDD (n = 127)

*Recurr (n)*, Number of recurrence after first episode in this IVDD (n = 67)

*Recurr (%)*, Percentage of recurrence after first episode of IVDD

*IVDS*, Intervertebral disc space

Comparing the first and second episodes of IVDD regarding their localization, only 55 patients could be evaluated since no information for the localization of the second episode was available for 12 patients (17.9%). 50% (10/20) of patients with a first episode of IVDD in the cervical spine showed signs of recurrence localized in the cervical spine again. The remaining 50% (10/20) of recurrence after cervical IVDD were localized in the thoracolumbar spine. In contrast, 62.9% (22/25) of patients with a first episode in the thoracolumbar vertebral segment had a second episode localized in the same segment, compared to 37.1% of patients (13/35) who showed recurrence in the cervical spine.

### Grades of severity of neurological signs

French Bulldogs with a first episode of IVDD in the cervicale spine were most often presented with hyperesthesia only (50.9% with grade 1). They showed a recurrence rate of 57% (15/26). By contrast, most dogs with thoracolumbar IVDD were presented with a neuro-grade 3. The recurrence rate for these patients was 63.3% (19/30). Nevertheless, there was no statistically significant association of neuro-severity grade and risk for recurrence of IVDD.

### Spinal malformations

In total, 46 patients with vertebral malformations were observed in 133 French Bulldogs (36.2%). Among the malformations, 44 wedge vertebra, 11 butterfly vertebra, and 9 thoracolumbar transition vertebrae were detected. In 16/46 patients (34.7%) more than one vertebral malformation was present on diagnostic imaging. 55.2% (42/76) of patients with thoracolumbar IVDD showed vertebral malformations compared to 7.8% (4/51) with cervical IVDD. There were only 18/127 (14.2%) French Bulldogs with vertebral body malformations that were less than 2 IVDS apart of the IVD affected at the first episode. 11/18 (61.1%) patients showed signs of recurrence. No evidence of an association between vertebral body malformations and recurrence of IVDD was found in this sample. In our study population only 58/127 patients (45.7%) had MRI of the complete thoracic spine. 68.9% (40/58) patients with a complete thoracic MRI showed vertebral malformations with a recurrence rate of 57.5% (23/40).

### Intervertebral disc degeneration (IVDd)

99/127 (77.9%) French bulldogs showed multiple degenerated intervertebral disc spaces at MRI examination when presenting with their first episode of IVDD. 50/67 (74.6%) patients with recurrence showed degenerated IVD. When considering all lesions (cervical and thoracolumbar), we did not find evidence of IVD degeneration to be associated with recurrence of IVDD.

### Intervertebral disc fenestration

Only 17/127 patients (11%) received additional disc fenestration during surgery of first episode of IVDD. In 13/17 patients (76.5%) additional fenestration was performed in the same intervertebral disc space, in 4/17 cases (23.5%) the fenestration was performed in the adjacent IVD and for 2 patients an additional approach was performed to fenestrate a distant IVD. In two patients more than one IVD were fenestrated. 41.1% (7/17) of patients with intervertebral disc fenestration showed signs of recurrence. There was no statistically significant influence on recurrence of IVDD.

## Discussion

We found that recurrence of IVDD in French Bulldogs is a common phenomenon in around 53% of patients previously presented with a first episode of IVDD. This is in agreement with a recent report showing a recurrence rate of 51% in this specific dog breed [14]. However, compared to this study, we examined a larger sample of patients and analyzed the relationship between different risk factors for Total-Recurrence as well as Cervical- and Thoracolumbar-Recurrence of IVDD separately, using logistic regression models. Our results indicate an association of age with Total-Recurrence and Cervical-Recurrence. More than 50% of the recurrences occurred within the first 12 month after the first episode of IVDD.

No evidence of an association was found between gender, body weight, localization of first episode, neurological severity grade and-recurrence of IVDD which is in accordance with observations of previous studies in different dog breeds [4, 5, 10–12, 15, 17, 21].

Contrary to these studies [4, 5, 12, 17, 22], a significant association between age and IVDD recurrence was found for French Bulldogs. Recurrence occurred significantly more often in French Bulldogs who had suffered from a first episode of IVDD at a young age (≤ 3 years) compared to dogs that were 6 years or older (≥ 6 years). Previous studies highlighted the fact that first episodes of IVDD occur earlier in French Bulldogs than in other dog breeds [2, 23]. This may be related to the chondrodystrophic characteristics of this breed and early-onset of disc degeneration [3, 24]. It was shown that the nucleus pulposus is already transformed from notochord-cells to chondrocyte-like cells at the age of 2–3 month which is earlier than in non-chondrodystrophic dog breeds [3, 25]. As found in other reports, vertebral body malformations and kyphosis accelerate the IVD degeneration in the adjacent IVDS [26, 27]. Therefore, we differentiated between the presence of vertebral malformations less or more than 2 IVDS apart from the site of the first IVDD. However, our results do not indicate any association of vertebral body malformations with recurrence of IVDD in both groups, which is in accordance with older studies [23, 27]. As limitation of our results, it has to be considered that we identified only 46/127 (36.2%) French Bulldogs with vertebral body malformations. By contrast, two recently published studies observed 87% [14] and 88% [27] of French Bulldogs, respectively, including only dogs with a MRI examination of the complete thoracic vertebral column. Since we additionally included cervical IVDD in our sample, only 46% (52/127) of our patients had an MRI examination of the complete thoracic vertebral column. When the patients with a MRI examination of the complete thoracic segment are examined separately in our sample, 68.9% (40/58) showed vertebral malformations in the thoracolumbar spine. Due to these inconsistencies, the results cannot be compared to the above-mentioned studies. Consequently, it has to be assumed that the MRI examination of the thoracic vertebral column is mandatory to evaluate vertebral malformations in French Bulldogs, as most of the vertebral malformations are located in the thoracic vertebral segment [26, 28, 29].

A possible reason for the lower number of IVDD cases for dogs older than five years (≥ 6 years) might be the reduced calcification of the nucleus pulposus. Disc calcification reaches a maximum at an age of 18–24 months [4, 30, 31], because calcified deposits are broken down by inflammatory cells [4, 31]. The calcification of the nucleus pulposus is part of degenerative changes, which indicates a complete disc degeneration and leads to a limitation of its shock absorbing effect [4, 30, 31], resulting in a higher risk of recurrence [24]. This is in accordance with older reports assessing disc calcifications evaluating radiographs for disc calcification in different chondrodystrophic dog breeds [4, 32]. A recent study assessing MRI images showed the same effects [33]. Compared to radiographic imaging, the MRI examination allows to detect earlier stages of disc degeneration, since changes can be detected before calcification occurs [33, 34]. Contrary to these results, we were not able to find an association between intervertebral disc degeneration and risk of recurrence by evaluating MRI images, however. The use of a two-grade classification in our study instead of the Pfirrmanns-grading scale might have influenced the results [19].

All patients in the current study were treated surgically. Prophylactic fenestration was performed in only 13.4% of patients in our study sample with a recurrence rate of 41.2%. Compared to several reports indicating that prophylactic disc fenestration decreases the risk of recurrence in dogs undergoing surgical treatment [4, 5, 35, 36], we were currently not able to detect a significant influence. As a possible explanation for the low number of patients with prophylactic disc fenestration in our sample it has to be considered that fenestration caudal to L3 is not routinely recommended due to post operative complications of iatrogenic trauma of closely related anatomic structures [10, 37]. L3-L4 was the most common affected IVDS in our sample. Further possible complications as hemorrhage of spinal artery/vein, introduction of further nucleus pulposus material in the vertebral canal, spinal nerve root trauma [36], vertebral instability [6] and prolonged anesthesia time [5]) might also have influenced the surgeons decisions to perform this additional procedure. Finally, the advantages of additional prophylactic disc fenestration seem to be still unclear for many surgeons. A recently published questionnaire-based study showed that only 55% of surgeons and 82% of neurologists use this technique routinely [35, 36].

Approximately 47% of the French Bulldogs in the present study suffered from a recurrence after a first episode of cervical IVDD (most often C3-C4). The thoracolumbar spine was even more frequently affected by approximately 57.7% of recurrences. This is in agreement with a previous report showing 48% recurrence in cervical and 53% recurrence in thoracolumbar spine in French Bulldogs [14]. Similarly, two other recent studies indicated a higher number of caudal thoracolumbar and lumbar IVDD in French Bulldogs due to the kyphotic position of the thoracal spine [23, 27]. It is suggested that the kyphotic position leads to abnormal biomechanical forces in the distant lumbar IVD, causing a higher risk for IVDD caudal to the thoracal spine [23, 27]. Since other dog breeds with similar curvature abnormalities as French Bulldogs do not show the same prevalence for lumbar IVDD, the relevance of the kyphotic position of the thoracal spine is still unclear [27]. However, the presence of kyphosis was not part of our examinations.

A further limitation of this study is that the follow up time was not equal for all patients. For some patients the follow up time was 6 months compared to others with 9 years. As most recurrences occurred within the first 12 months after the first episode of IVDD, the authors suggest that the recurrence of IVDD would be even higher if all patients received a minimal follow up time of 2–3 years.

An additional limitation of this study is the potential selection bias due to telephone follow-up. Patients, for whom owners reported episodes of back pain with subsequent improvement with analgetic-treatment only were also classified as recurrence. However, for almost half of all enrolled subjects with a recurrence, no MRI was performed. In previous studies, patients showing only signs of hyperesthesia were excluded. In these studies, the recurrence rates were between 15.8% and 19.3% [4, 5, 15] focusing on thoracolumbar IVDD only. By contrast, the recent study of Kerr et al. (2021) showed similar recurrence rates in French Bulldogs (51%) as we did, but the rate of MRI-confirmed recurrences was even lower (37%) compared to our results (46.2%) [14]. It can therefore be assumed that the inclusion of patients with hyperesthesia only without further diagnostic imaging might lead to a potential overestimation of recurrences. However, since French Bulldogs suffer more often from cervicale IVDD than other dog breeds [2], they are particularly predisposed to be presented with hyperesthesia only (severity grade 1). A possible explanation might be the relative dimension of the spinal canal which is wider in the cervical than in the thoracolumbar spine and therefore less likely to be compressed severely [8]. Additionally, the discs in the cervical area are generally less calcified and may elicit less inflammation and damage to the spinal cord [24, 38]. Therefore, it was reasonable for the authors to include also patients with suspicion of recurrence with hyperesthesia only without neurological deficits and no further diagnostic imaging in this study. Ideally, to allow a precise statement of the recurrence rate of IVDD in French Bulldogs, a prospective study will have to be conducted in which all patients with clinical signs attributable to recurrences of IVDD have to undergo an MRI examination.

## Conclusions

Recurrence of IVDD can be expected in more than half of French Bulldogs affected by IVDD. Especially young French Bulldogs are prone for recurrence in the cervical spine. Almost every fourth patient with IVDD suffers from a recurrence within 12 months. Future dog owners should be informed about the risk of IVDD and the early onset of recurrences in French Bulldogs.

## Data Availability

The datasets used and analyzed during the current study are available from the corresponding author on reasonable request.

## Abbreviations

IVDD: Intervertebral disc disease
IVDd: Intervertebral disc degeneration
IVDS: Interverterbal disc space
IVD: Intervertebral disc
MRI: Magnetic resonance imaging
